# Perceived health and work-environment related problems and associated subjective production loss in an academic population

**DOI:** 10.1186/s12889-018-5154-x

**Published:** 2018-02-14

**Authors:** Malin Lohela-Karlsson, Lotta Nybergh, Irene Jensen

**Affiliations:** Unit of Intervention and Implementation Research for Worker Health, Institute of Environmental Medicine (IMM), Karolinska Institutet, 171 77 Stockholm, SE Sweden

**Keywords:** Health problems, Work environment, Production loss, Academic employees, Gender

## Abstract

**Background:**

The aim was to investigate the prevalence of health problems and work environment problems and how these are associated with subjective production loss among women and men at an academic workplace. An additional aim was to investigate whether there were differences between women and men according to age group, years at current workplace, academic rank or managerial position.

**Methods:**

A questionnaire was sent in 2011 to all employees at a Swedish university (*n* = 5144). Only researchers and teachers were included in the study (*n* = 3207). Spearman correlations were performed to investigate differences in health and work environment problems. Employees who reported having experienced work environment or health problems in the previous seven days (*n* = 1475) were included in the analyses in order to investigate differences in subjective production loss. This was done using Student’s *t*-test, One-way Anova and generalized linear models.

**Results:**

The response rate was 63% (*n* = 2022). A total of 819 academic staff (40% of the population) reported experiencing either health problems, work environment problems or both during the previous seven days. The prevalence of health problems only or a combination of work environment and health problems was higher among women than men (*p*-value ˂0.05). This was especially the case for younger women, those in lower academic positions and those who had worked for fewer years at their current workplace. No difference was found for work environment problems. The majority of the employees who reported problems said that these problems affected their ability to perform at work (84–99%). The average production loss varied between 31 and 42% depending on the type of problem. Production loss due to health-related and work-environment related problems was highest among junior researchers and managers. No significant difference between men and women was found in the level of production loss.

**Conclusion:**

Subjective production loss in academia can be associated with health and work- environment problems. These losses appear similar for women and men even though younger female academics, women in lower academic ranks and those with fewer years of employment in their current workplace report a higher prevalence of health problems and combined work-environment and health problems than men.

## Background

In academia, productivity is measured by such factors as published papers and success in obtaining funding. Publications are recognized as a key indicator for measuring scientific productivity. A researcher’s success in publishing papers and attracting research grants is therefore crucial for his or her future academic career. Researchers with short publication lists and few papers published in high impact journals can have difficulty in securing research grants. As research is often financed by research grants, the failure to obtain grants tends to force the researcher to leave the academic world. Success, therefore, depends on being able to get published from early on in the career.

Several studies that compare the publication rates between female and male researchers demonstrate that women publish less than men [1, 2]. Some studies find these differences to be especially pronounced in resource-demanding areas of research, although the discrepancy continues across disciplines [3]. This discrepancy has been found to emerge over time, starting as early as in the first two years after completing a doctorate [4], which are often a critical time for the future academic career.

Several explanations for the differences in scientific productivity between women and men have been proposed. For example, it has been argued that women receive academic positions that have less potential for future promotion and tenure [5]. It has also been suggested that they do more administrative work and are more involved in teaching than men, which leaves less time for research and therefore publication [6, 7]. Furthermore, it has been found that the explanatory power of sex decreases or loses its significance when academic rank [8–12], academic discipline [2, 8, 9, 12, 13], marital status, number of children [12] and time set aside for research [9] are controlled for. For example, female professors spend more time on administration than male professors, while female assistant professors spend significantly fewer hours on research than male assistant professors [14]. Moreover, men have been found to adopt more successful career strategies for research productivity, such as specializing in one subject instead of diversifying [15]. Reasons have also been sought in the work-family balance and whether women’s higher share of child and household-related responsibilities might add to the lower production rates. However, the evidence for this is inconclusive [16–18]. Finally, one recent study found that the differences in productivity between women and men disappear in the younger generation of researchers [19], raising the question of whether the gap might be narrowing.

Studies have also investigated the impact of workplace factors. It has been shown that scientific productivity is indeed related to both individual and workplace factors [11, 20, 21]. In particular, a supportive leadership with high scientific competence which puts a premium on scientific productivity, a good communicative group climate, and a transformative research group (i.e. allowing new people to enter the group) has been found to be associated with high scientific productivity. Having an external network, the necessary resources, clear goals and good communication are also of importance for high scientific productivity [11]. In contrast, a poor work environment, excessive workload and lack of institutional influence have been identified as barriers to scientific productivity [22]. Men are more likely than women to report a supportive and satisfying work environment in which colleagues value each other’s contributions. This, in turn, is associated with higher levels of scientific productivity [12]. Female academics also experience more discrimination than men, which impedes productivity levels [12]. A study conducted in Sweden found that a high level of control over one’s work was associated with high productivity among male but not among female academics, whereas high levels of exhaustion were associated with a negative impact on productivity among women but not among men [23].

In a previous study of academic employees experiencing work environment problems it was found that unfair leadership, inequality and role conflicts were associated with higher levels of subjective production loss [24]. Work environment problems and presenteeism (going to work while sick) [25], have previously been shown to be related to subjective production loss [26–28], resulting in reduced productivity for organizations. Production loss is a consequence of presenteeism and work environment-related problems and can be defined as the difference between an employee’s regular performance and his or her performance while affected by the problem [28]. One possible explanation for the male-female differences in scientific productivity could be that men and women experience different levels of work environment problems or health problems, since it is these problems that tend to affect the ability to perform at work. However, we know very little about whether female and male academic staff experience different levels of work environment or health problems and how this may affect their productivity. Similarly, little is known about whether there are differences in how academic staff of different rank experience the work environment or the experience of having health problems, and whether this affects their productivity.

The overall aims of the present study were to investigate the prevalence of perceived health and work environment problems and how these are associated with subjective production loss among women and men at an academic workplace. An additional aim was to investigate whether there were any differences in the prevalence of perceived health and work environment problems and subjective production loss between women and men according to age group, years of employment at their current workplace, academic rank or managerial position.

## Methods

In the autumn of 2011, a cross-sectional study using a validated questionnaire was conducted among employees at a Swedish university. The aim was to assess and improve the work environment by assessing social and organizational factors and subjective production loss. All staff who had been employed at the university on at least 50% of a full time post and for a duration of at least six months at the time of the assessment, were invited to participate (*n* = 5143). The majority (*n* = 3207) were academic staff, i.e. researchers or teaching staff. The invitation was sent out by email and was followed by two reminders. The responses were anonymous for the employer and were forwarded directly to the research team. Participation was voluntary and written informed consent was obtained from all respondents. A total of 3515 employees responded. This specific study only includes employees employed as academic staff (*n* = 2022), with a response rate of 63%. Administrative staff were excluded from this study.

### Measurements

The questionnaire included the following items:

- Health problems

All employees were asked whether they had decided to go to work despite perceiving health problems in the previous seven days. Health problems were defined as any physical or mental health problem or symptom. The response options were yes/no. Employees who answered “yes” were defined as having health problems.

- Work environment problems

Employees were asked whether they had experienced any work environment problems in the previous seven days. Work environment problems were defined as any physical, psychological or social problems that resulted from the work environment. Response options were yes/no. Employees who answered “yes” were defined as having work environment problems.

- Health-related production loss

Those who answered yes to the question about health problems were asked whether their health problems had affected their performance while at work during the previous seven days. Respondents were asked to rate how much their performance had been affected on a scale from 0 to 10, where 0 = no effect on my work and 10 = completely prevented me from working. This question has been shown to be valid and reliable in a Swedish context [29, 30]. The rated reduction in performance was used to capture the subjective production loss. This scale was converted from 0 to 10 to 0–100 to capture the percentage loss of work time.

- Work environment-related production loss

Those who reported work environment problems were asked to quantify how much these problems had affected their performance at work. The scale ranged from 0 to 10 where 0 = no effect on my work and 10 = completely prevented me from working. The item has been shown to be valid and reliable in a Swedish population [28, 30]. The rated reduction in performance was used to capture the subjective production loss. This scale was converted from 0 to 10 to 0–100 to capture the percentage loss of work time.

#### Socioeconomic data

The employees were divided into the following occupational categories obtained from the university employment register: professors, senior researchers (associate professors, researchers), junior researchers (post docs or assistant professors), PhD students and teachers. The category teacher consisted of employees employed as junior and senior lecturers.

Information about managerial position, age and years at the current workplace was also collected. Managerial position had the response options – yes/no. Age was divided into the categories ≤29, 30–39, 40–49, 50–59 and ≥60 years. Years at current work place was divided into the categories: < 1 year, 1–2 years, 3–5 years, 6–10 years and > 10 years.

### Statistical analysis

The prevalence of the various problems was calculated from all responding academic employees (*n* = 2022). Statistical differences between groups were tested for using Spearman correlation. To analyse production loss, the employees were divided into three categories based on their responses to the questions about health or work environment problems. These categories were: 1) yes, health problems only, 2) yes, work environment problems only and 3) yes, combined health and work environment problems. Employees responding “no” to both types of problems were excluded from this part of the study. The decision to divide the study population in this way was based upon a previous study showing that employees experiencing both types of problem are worse off in terms of how they perceive the work environment as well as their health and need to be studied independently [28]. The three categories were analyzed separately.

Potential differences between men and women in health problems, work environment problems or both according to age, rank, managerial position and years at current workplace were tested for using Spearman correlation. The average level of production loss caused by each problem was then calculated and significant differences between groups were tested using Student’s *t*-test and One-way Anova. Generalized linear models were used to evaluate differences in production loss for men and women by age, rank, managerial position and years at the current workplace. All the analyses were performed with SPSS version 20.

## Results

### Prevalence of perceived health and work environment problems

A total of 819 academic employees (40% of the population) reported having experienced either health problems, work environment- problems or both during the previous seven days. The prevalence of the three problems was 21%, 8%, and 11% respectively (Table 1). The prevalence of health problems was significantly higher among women than men (*p*-value < 0.05). The prevalence of experiencing both kinds of problems during the previous seven days was significantly higher for women than for men (*p*-value < 0.05). Significant differences were also found between rank (*p*-value < 0.05). For example, more senior researchers were less likely to report a combination of problems than were junior researchers. The highest prevalence of combined problems was found among teachers.

**Table 1:** Prevalence of perceived health problems, work environment problems and a combination of both problems in an academic population

	Total population (*n* = 2022)	Health problems (*n* = 433)	*p*-value	Work environment problems (*n* = 170)	*p*-value	Both health and work environment problems (*n* = 216)	*p*-value
Total, n (%)	2022	433 (21)		170 (8)		216 (11)	
Sex, n (%)			**0.01** ^**1**^		0.25^1^		**0.00** ^**1**^
Men	881	162 (18)		67 (8)		65 (7)	
Women	1141	271 (24)		103 (9)		151 (13)	
Age, mean (sd)	41 (12)	41 (12)	0.72^1^	41 (11)	0.88^1^	40 (10)	0.12^1^
Age, n (%)							
≤29	328	71 (22)		28 (9)		36 (11)	
30–39	748	167 (22)		63 (8)		84 (11)	
40–49	423	86 (20)		35 (8)		53 (13)	
50–59	320	57 (18)		29 (9)		36 (11)	
≥60	203	52 (26)		15 (7)		7 (3)	
Manager, n (%)			0.30^1^		0.15^1^		0.32^1^
Yes	525	104 (20)		52 (10)		50 (10)	
No	1497	329 (22)		118 (8)		166 (11)	
Rank, n (%)			0.47^1^		0.79^1^		**0.01** ^**1**^
Professors	247	53 (21)		18 (6)		11 (4)	
Senior researcher	637	150 (20)		51 (17)		50 (9)	
Junior researcher	130	23 (18)		16 (12)		16 (12)	
PhD student	731	149 (20)		57 (8)		95 (13)	
Teacher	277	58 (21)		28 (10)		44 (16)	
Years at current workplace, n (%)			0.15^1^		0.48^1^		0.24^1^
< 1 year	113	23 (20)		7 (6)		16 (14)	
1–2 years	418	102 (24)		32 (8)		43 (10)	
3–5 years	665	137 (21)		60 (9)		77 (12)	
6–10 years	339	82 (24)		29 (9)		35 (10)	
> 10 years	487	89 (18)		42 (9)		45 (9)	
Production loss due to the problem							
Yes	–	364 (84)	–	158 (93)	–	213 (99)	–
No	–	69 (16)		10 (6)		3 (1)	
Missing				2 (1)			

^1^Spearman correlation

The majority of those who reported problems said that these affected their ability to perform at work. Almost all employees experiencing work environment problems and combined problems (93% and 99% respectively) reported that their work performance was affected. This consequence was, however, smaller in the group which reported health problems only (84%).

Table 2 presents the prevalence of the different types of problems by gender. Health problems were more common among younger women (up to the age of 49) than among men in same age group (*p*-value < 0.01). Health problems were also more prevalent among female PhD students, junior and senior researchers and teachers than among men in the same positions (*p*-value < 0.01). They were also more prevalent among women than among men in non-managerial positions (*p*-value < 0.01) as well as among those with 1–10 years’ employment at their current workplace (*p*-value < 0.01).

**Table 2:** Number of employees perceiving health problems, work environment or both types of problem, presented for men and women separately and by age, rank, managerial position and years at current workplace

	Total population (*n* = 2022)		Health problems (*n* = 433)			Work environment problems (*n* = 170)			Both types of problems (*n* = 216)		
	Men	Women	Men	Women	*p*-value^1^	Men	Women	*p*-value^1^	Men	Women	*p*-value^1^
Total, n (%)	881	1141	162 (18)	271 (24)		67 (8)	103 (9)		68 (8)	151 (13)	
Age, n (%)					**0.01**			0.29			**0.00**
−29	118	210	17 (14)	54 (26)		9 (7)	19 (9)		8 (7)	28 (13)	
30–39	302	446	55 (18)	112 (25)		18 (6)	45 (10)		27 (9)	57 (13)	
40–49	196	227	35 (18)	51 (22)		19 (10)	16 (7)		19 (10)	37 (16)	
50–59	148	172	25 (17)	32 (17)		12 (8)	17 (10)		9 (6)	27 (16)	
60+	117	86	30 (26)	22 (26)		9 (8)	6 (7)		5 (4)	2 (2)	
Rank, n (%)					**0.00**			0.23			**0.01**
Professor	178	69	38 (21)	15 (22)		13 (7)	5 (7)		7 (4)	4 (6)	
Teacher	85	192	14 (16)	44 (23)		8 (9)	20 (10)		9 (11)	35 (18)	
Researcher	305	332	65 (21)	85 (26)		23 (8)	28 (8)		18 (6)	32 (10)	
Junior researcher	57	73	8 (14)	15 (21)		7 (12)	9 (12)		7 (12)	9 (12)	
PhD student	256	475	37 (14)	112 (24)		16 (6)	41 (9)		24 (9)	71 (15)	
Manager, n (%)					**0.00**			0.66			**0.00**
Yes	317	208	61 (19)	43 (21)		32 (10)	20 (9)		27 (9)	23 (11)	
No	564	933	101 (18)	228 (24)		35 (6)	83 (9)		38 (7)	128 (14)	
Years at current workplace, n (%)					**0.00**			0.50			**0.00**
< 1 year	46	67	10 (22)	13 (19)		3 (7)	4 (6)		4 (9)	12 (18)	
1–2 years	173	245	35 (20)	67 (27)		8 (5)	24 (10)		9 (5)	34 (14)	
3–5 years	273	392	45 (16)	92 (23)		22 (8)	38 (10)		24 (9)	53 (14)	
6–10 years	130	209	26 (20)	56 (27)		15 (12)	14 (7)		6 (5)	29 (14)	
> 10 years	259	228	46 (18)	43 (19)		19 (7)	23 (10)		22 (8)	23 (10)	

^1^Spearman correlation

Differences between women and men in the experience of work environment problems in the different categories were not shown to be statistically significant.

Experiencing combined problems was more common among women than among men. The differences were found in all age groups below the age of 60 (*p*-value < 0.01), among teachers, senior researchers and PhD students (*p*-value < 0.01), female non-managers (*p*-value < 0.01) and among those who had worked at their current workplace for 10 years or less (*p*-value < 0.01).

### Average level of subjective production loss

Table 3 presents the average level of subjective production loss for employees according to the different categories. The average health-related production loss differed significantly between age groups, with the lowest levels among the oldest employees aged 50 or above (*p*-value = 0.02). The highest level was found in the age group 40–49. The level of subjective work environment-related production loss did not differ significantly between any of the investigated categories. Significant differences in the average level of production loss among those experiencing both kinds of problems were found between those in managerial and non-managerial positions (*p*-value = 0.02) as well as among different ranks (*p*-value = 0.04). The highest level was found among the teaching staff and the lowest level among the professors. On a scale of 0–100, researchers (both senior and junior and PhD students) all reported a level of production loss of 40% or higher.

**Table 3:** Average level of subjective production loss in a working population divided into different categories

	Health-related production loss^1^(*n* = 433)		Work environment-related production loss^1^(*n* = 168)		Both health and work environment-related production loss^1^ (*n* = 216)	
	Mean (sd)	*p*-value	Mean (sd)	*p*-value	Mean (sd)	*p*-value
Total	31 (26)		42 (26)		41 (23)	
Sex		0.07^2^		0.91^2^		0.27^2^
Men	28 (25)		41 (26)		38 (20)	
Women	33 (27)		42 (25)		42 (24)	
Age		**0.02** ^**3**^		0.34^3^		0.19^3^
−29	34 (23)		43 (26)		38 (20)	
30–39	33 (30)		46 (27)		44 (24)	
40–49	36 (28)		37 (25)		41 (22)	
50–59	25 (21)		35 (24)		40 (24)	
60+	23 (21)		43 (21)		24 (16)	
Manager		0.75^2^		0.53^2^		**0.01** ^**2**^
Yes	31 (31)		40 (25)		33 (19)	
No	31 (27)		42 (25)		43 (24)	
Rank						
Professor	25 (21)	0.08^3^	36 (22)	0.36^3^	21 (15)	**0.04** ^3^
Senior researcher	34 (30)		42 (26)		41 (24)	
Junior researcher	30 (24)		43 (22)		40 (23)	
PhD student	33 (26)		45 (28)		41 (22)	
Teacher	26 (22)		35 (21)		45 (24)	
Years at current workplace		0.32^3^		0.13^3^		0.12^3^
< 1 year	36 (32)		45 (16)		41 (22)	
1–2 years	33 (25)		41 (29)		41 (25)	
3–5 years	33 (27)		48 (26)		43 (20)	
6–10 years	29 (26)		39 (23)		46 (26)	
> 10 years	27 (24)		34 (22)		33 (21)	

^1^Production loss was assessed on a scale ranging from 0 to 100

^2^Students *t*-test

^3^Oneway Anova

General linear models were used to test for potential differences in subjective production loss for men and women in the different categories (Table 4). The average level of production loss for employees with health problems was lower for workers aged 60 or older than for employees aged 29 or younger (β = −11.0, CI = −20.2--1.9). The difference remained when sex was controlled for (β = −9.8, CI = −19.1--0.5). A significantly lower level of production loss was also found for professors compared to PhD students in the group experiencing health problems (β = −8.4, CI = −16.4- -0.3). No significant difference remained when sex was controlled for.

**Table 4:** Associations between age, rank, being a manager and years at the current workplace and subjective production loss for men and women

	Health-related production loss (*n* = 433)		Work environment-related production loss (*n* = 168)		Both health- and work environment-related production loss (*n* = 216)	
	Β (CI)^1^	Β (CI)^2^	Β (CI)^1^	Β (CI)^2^	Β (CI)^1^	Β (CI)^2^
Age						
60+	**−11.0 (− 20.2--1.9)**	**−9.8 (− 19.1--0.5)**	−0.6 (− 16.1–15.0)	−0.8 (− 16.5–15.0)	−14.6 (−32.8–3.5)	−13.1 (−31.5–5.3)
50–59	−8.1 (−17.0–0.8)	−7.4 (−16.3–1.6)	−8.2 (−21.2–4.8)	8.3 (−21.3–4.8)	1.8 (− 8.6–12.2)	1.9 (− 8.5–12.2)
40–49	2.2 (−5.9–10.2)	2.8 (−53–10.9)	−5.8 (−18.1–6.6)	−6.0 (−18.4–6.5)	2.5 (− 7.0–12.0)	2.7 (−6.8–12.2)
30–39	−1.0 (− 8.1–6.1)	− 0.7 (− 7.8–6.4)	2.4 (− 8.7–13.5)	2.5 (− 8.6–13.6)	5.7 (−3.0–14.5)	6.0 (−2.7–14.8)
− 29	0	0	0	0	0	0
Rank						
Professor	**−8.4 (− 16.4- -0.3)**	−6.3 (− 14.8–2.1)	−9.6 (−23.0–3.9)	−9.7 (− 23.6–4.2)	**− 20.8 (− 34.7--7.0)**	**−20.1 (− 34.2--6.0)**
Teacher	−7.6 (− 15.4–0.2)	−7.6 (− 15.4–0.2)	−10.8 (− 22.1–0.5)	−10.8 (−22.1–0.5)	3.5 (−4.4–11.4)	3.4 (−4.5–11.4)
Researcher	0.8 (−5.1–6.6)	1.6 (−4.3–7.5)	−3.0 (−12.5–6.4)	−3.0 (−12.7–6.5)	0.0 (− 7.6–7.6)	0.2 (− 7.4–7.9)
Junior researcher	−2.7 (−13.9–8.6)	−2.2 (− 13.5–9.1)	−2.9 (− 16.7–10.9)	− 3.0 (− 16.8–10.9)	−1.0 (− 12.7–10.8)	−0.6 (− 12.4–11.1)
PhD student	0	0	0	0	0	0
Manager						
Yes	0.9 (− 4.8–6.6)	−0.4 (− 6.3–5.5)	2.7 (− 5.6–11.0)	2.8 (− 5.9–11.5)	**9.8 (2.8–16.9)**	**9.4 (2.1–16.8)**
No	0	0	0	0	0	0
Years at the current workplace						
< 1 year	9.1 (−2.7–21.0)	8.8 (−3.1–20.6)	10.6 (−10.5–31.7)	10.6 (− 10.5–31.7)	8.0 (−4.7–20.8)	7.6 (−5.3–20.4)
1–2 years	5.6 (− 1.8–12.9)	4.8 (− 2.5–12.2)	6.9 (− 4.5–18.3)	6.9 (− 4.6–18.4)	7.9 (−1.4–17.3)	7.4 (− 2.1–16.9)
3–5 years	6.3 (− 0.7–13.1)	5.4 (− 1.5–12.4)	**13.1 (3.3–22.9)**	**13.1 (3.3–22.9)**	**9.5 (1.3–17.7)**	**9.2 (0.9–17.5)**
6–10 years	2.3 (− 5.5–10.0)	1.4 (− 6.4–9.2)	4.2 (− 7.5–16.0)	4.2 (− 7.5–16.0)	**12.8 (2.9–22.7)**	**12.2 (2.1–22.2)**
> 10 years	0	0	0	0	0	0

Presented separately for groups with health-related, work environment-related or both health and work environment-related production loss

^1^Unadjusted

^2^Adjusted for sex

*Statistically significant associations in the table are marked with bold text*

Employees who had worked at their current workplace for 3–5 years reported a 13% higher work environment-related production loss than those who had been working there for more than 10 years (β =13.1, CI = 3.3–22.9). No differences were found between men and women.

Among the academic staff experiencing both health and work environment problems, professors reported an on average 20% lower production loss than did PhD students (β = − 20.8, CI = −34.7--7.0). Higher levels of loss were found for managers than for non-managers (β = 9.8, CI = 2.8–16.9) and for staff employed for 3–5 (β = 9.5, CI = 1.3–17.7) and 6–10 years (β = 12.8, CI = 2.9–22.7) compared to those who had been working for more than 10 years. No differences were found between men and women.

## Discussion

This study investigated the prevalence of perceived work environment problems and health-related problems among women and men at an academic workplace, as well as the corresponding subjective production loss. The results showed that 40% of the employees in this population had experienced at least one of these problems during the seven days prior to the study. The prevalence of health problems and combined problems was higher among women than men. However, there was no difference in the level of subjective production loss reported by the men and the women who were affected by these problems. Some differences in the reported levels of production loss for the problems under study were observed for other reasons than gender. Production loss could namely vary according to type of research position, number of years at current workplace and whether or not a managerial position was held. To the best of our knowledge, this is the first study investigating the prevalence and impact of health and work environment problems among academic employees.

While previous studies have shown that older employees experience more health problems than their younger counterparts, which results in a variety of consequences such as sickness absence [31, 32], this was only partly supported in this study. No significant differences in health problems were found between the different age groups, a finding supported by Robertson et al. [33]. Moreover, our results demonstrated an unexpected pattern which indicates that subjective production loss among those experiencing health problems was lowest among the oldest employees. This might be an expression of the “healthy worker effect” [34], according to which those over 60 years of age experiencing with health problems which make it difficucult to maintain normal performance levels had already left the organization. Another possible explanation is that the finding may be related to the type of work done by the oldest group of academic staff or their length of experience. For example, professors were found to report significantly less production loss than less senior academic staff, even when they reported health problems. An explanation for this might be that professors are more experienced than academic staff at the lower ranks. For example, writing articles, which is an important measure of research productivity, becomes faster with experience, which could affect how professors rate the level of production loss when they have perceived health problems. In addition, the work of professors often differs somewhat from that of younger, less experienced staff. Instead of writing the articles themselves, professors often supervise their younger colleguages and could therefore be involved in several manuscripts at the same time, resulting in high productivity rates. The production loss measure is subjective and asks people to rate their performance in relation to what they would have produced if they had not had health problems. Normal performance is therefore subjective and might be related to individual demands and expectations. A possible explanation for the difference between professors and lower-rank academic staff could be that the former are better able to adjust to demands and expectations that are achievable. It could also be that they are more likely to recognise that the inspiration behind good research is necessarily episodic and can come when least expected. For this reason they may be less concerned about productivity loss arising from health problems.

### Differences between women and men

In the current study, women reported a higher prevalence of health problems and combined problems than men. This was especially the case for younger women, those in lower academic positions and those who had worked at their current workplace for the fewest years. One possible explanation is that women who are younger and in more junior positions are likely also to have young children. Given the fact that women are also more likely than men to be primary caregivers, these circumstances may impose a greater strain on their health. However, previous studies of this subject are inconclusive [16–18]. An additional explanation for these findings may be the prevailing gender norms in academia, which have been found to favor men and hinder women with regard to establishing their authority, advancing their careers, securing lead authorship in publications and combining family with working life [35–37]. Previous studies have found that female academics struggle with gender bias, which could affect how they perceive their health and work environment. One might hypothesize that similar conditions may especially be found in the group that experienced a combination of health and work environment problems, which is the group for which the largest sex differences were found. There is a need for further studies into the consequences of gender discrimination for perceived work-environment and health problems.

Interestingly, the sex differences in perceived health and work environment problems did not translate into similar sex differences in subjective production loss at work. Rather, women and men reported similar levels of production loss as a consequence of the abovementioned problems. Even though there is a difference between the outcome measures of subjective production loss and objective productivity (e.g. number of publications), the finding that women experience more health problems than men, which in turn could affect women’s ability to maintain the same productivity rate as men, could be an additional explanation for the previously identified differences in productivity [1].This difference was especially found in the group of younger women in more junior positions, which might be an additional explanation for why women’s research productivity lags behind that of their male counterparts early in their careers. Health problems result in a production loss which potentially affects both the quality and the quantity of their scientific productivity. The fact that female researchers report more health problems than men might also explain why fewer female than male researchers become professors: health problems may be forcing them out of academic life. The results also show that employees experiencing these problems report similar levels of production loss irrespective of whether they are male or female. It is also possible that women and men evaluate perceived production loss differently, with either one of the sexes over- or underestimating the effects of health and work-environment problems on their work performance. It would have been interesting to investigate whether the self-reported production loss item would conform to an objective measure of the same or whether there might be discrepancies that could be gender-skewed. Future studies investigating the differences in objective productivity between women and men are suggested to account for potential differences in health status.

### Limitations

This study uses self-reported data, which is often seen as a limitation. However, self-reported measures have previously been shown to be reliable and valid. For example, the importance of self-rated health has been shown to be an independent predictor of future mortality [38]. Moreover, self-reported measures are a convenient and inexpensive way to assess the prevalence of health-problems among large populations in an organization. Work-environment problems, especially psychosocial ones, are difficult to measure objectively as people tend to perceive situations in the work environment differently. A situation perceived as problematic by one employee might not be perceived the same way by someone else. For this reason, a self-reported measure is relevant when the aim is to gain an overview of the prevalence of work-environment and health problems.

Nevertheless, constructions of masculinity and femininity may affect self-reporting behaviors in different ways for women and men. It is possible, for example, that men are less willing to disclose health-related issues because of social norms that construct men as the stronger sex [39]. Furthermore, there may be a selection of respondents, in that those with more health- or work-related problems may potentially be more willing to complete a survey on this topic than those who have fewer such problems. The prevalence of these issues may thus possibly be over-estimated. Future studies investigating this issue in relation to academic performance would be welcome.

Another limitation of the study might be the measure and interpretation of production loss. As is the case with work-environment problems, employees may be affected differently by the problems they perceive as being related to health or the work environment. For this reason, collecting self-reported data is a convenient way to gather information about these problems and how they affect the ability to perform at work. The validity of the measure of subjective production loss depends on validation against objective data on production. This is a general limitation for almost all instruments developed to measure subjective production loss [40]. However, validations using objective measures have been performed on one measure of subjective production loss and were demonstrated to show good validity in some groups of workers [41].

This study investigated the direct association between health and/or work environment problems and subjective production loss. Since the study used a cross-sectional design it was not possible to investigate the causal effect, i.e. whether a detoriation in health results in a drop in productivity, or whether a perceived loss in productivity among healthy employees resulted in greater stress and then caused deterioration in health. It is also possible that health problems cause production loss, which in turn causes stress that results in reduced productivity. This cause/effect relationship needs to be investigated further in order to increase our understanding of differences in scientific productivity in general, as well as between women and men.

The results of this study offer an additional explanation for why women’s research productivity is lower than that of their male counterparts. The reason for why younger women in lower academic ranks perceive more health problems than men needs to be investigated further. This knowledge, together with previous studies in the area, can help to improve opportunities for women to remain in academia and reach more senior positions.

## Conclusion

The results show that female academic employees reported a higher prevalence of health problems and a combination of work environment and health problems than men. This was especially the case for younger women, those in lower academic ranks and those with fewer years at their current workplace. However, these differences were not translated into similar sex differences in perceived performance loss while at work. This suggests that the previously identified differences in productivity can to some extent be explained by younger women in more junior positions experiencing more health problems than men. This might, in turn, help to explain why women’s research productivity lags behind that of male researchers early in their careers.
